# Supplementary material for: Meta-Analysis and Gene Set Enrichment Relative to ER Status Reveal Elevated Activity of MYC and E2F in the “Basal” Breast Cancer Subgroup
Source: PLoS One. 2009 Mar 9;4(3):e4710. doi: 10.1371/journal.pone.0004710 (PMC2650420; doi:10.1371/journal.pone.0004710)
Supplement: Dataset S1 — GSEA results from first screen. The file contains a zipped archive of the HTML results of the second GSEA screen. Reports for enriched or depleted datasets may be accessed from the “index.html” link within the my_analysis.GseaPreranked.1219975950625.rpt.zip ZIP archive. Enrichment plots are available for each of the 50 most enriched and depleted gene sets. (13.32 MB ZIP) [file pone.0004710.s001.zip › my_analysis.GseaPreranked.1219975950625.rpt.zip!//CHR17Q21.html]

Details for gene set CHR17Q21[GSEA]

|  || Dataset | zSco.ERneg.to.ERpos\_collapsed |
| Phenotype | NoPhenotypeAvailable |
| Upregulated in class | na\_neg |
| GeneSet | CHR17Q21 |
| Enrichment Score (ES) | -0.38629866 |
| Normalized Enrichment Score (NES) | -1.9668964 |
| Nominal p-value | 0.0 |
| FDR q-value | 0.010103239 |
| FWER p-Value | 0.253 |
Table: GSEA Results Summary

  

Fig 1: Enrichment plot: CHR17Q21      
 Profile of the Running ES Score & Positions of GeneSet Members on the Rank Ordered List

  

| PROBE | GENE SYMBOL | GENE\_TITLE | RANK IN GENE LIST | RANK METRIC SCORE | RUNNING ES | CORE ENRICHMENT || 1 | KRT16 |  |  | 274 | 5.009 | -0.0037 | No |
| 2 | LOC146909 |  |  | 317 | 4.875 | 0.0097 | No |
| 3 | GJA7 |  |  | 853 | 3.702 | -0.0184 | No |
| 4 | CNP |  |  | 1169 | 3.274 | -0.0312 | No |
| 5 | HOXB9 |  |  | 1179 | 3.261 | -0.0207 | No |
| 6 | TBX21 |  |  | 1851 | 2.648 | -0.0627 | No |
| 7 | CCL4 |  |  | 1943 | 2.576 | -0.0609 | No |
| 8 | ITGB3 |  |  | 2000 | 2.531 | -0.0565 | No |
| 9 | FLJ10120 |  |  | 2133 | 2.430 | -0.0582 | No |
| 10 | SDK2 |  |  | 2260 | 2.344 | -0.0598 | No |
| 11 | C17ORF53 |  |  | 2360 | 2.281 | -0.0596 | No |
| 12 | KRT13 |  |  | 2625 | 2.131 | -0.0724 | No |
| 13 | KRT15 |  |  | 2634 | 2.127 | -0.0657 | No |
| 14 | KRT23 |  |  | 2670 | 2.106 | -0.0612 | No |
| 15 | AOC2 |  |  | 2776 | 2.053 | -0.0622 | No |
| 16 | MYOHD1 |  |  | 3008 | 1.927 | -0.0732 | No |
| 17 | KPNB1 |  |  | 3125 | 1.876 | -0.0756 | No |
| 18 | KRT9 |  |  | 3150 | 1.865 | -0.0710 | No |
| 19 | CDC6 |  |  | 3185 | 1.847 | -0.0673 | No |
| 20 | KCNH4 |  |  | 3257 | 1.815 | -0.0665 | No |
| 21 | C1QL1 |  |  | 3280 | 1.803 | -0.0621 | No |
| 22 | ITGA2B |  |  | 3505 | 1.705 | -0.0733 | No |
| 23 | COL1A1 |  |  | 3516 | 1.699 | -0.0682 | No |
| 24 | HOXB1 |  |  | 3708 | 1.619 | -0.0772 | No |
| 25 | TOP2A |  |  | 3735 | 1.610 | -0.0737 | No |
| 26 | CCL11 |  |  | 3899 | 1.554 | -0.0808 | No |
| 27 | SPAG9 |  |  | 3926 | 1.544 | -0.0775 | No |
| 28 | GIP |  |  | 3930 | 1.542 | -0.0725 | No |
| 29 | ETV4 |  |  | 4095 | 1.475 | -0.0799 | No |
| 30 | MPO |  |  | 4106 | 1.469 | -0.0757 | No |
| 31 | DLX4 |  |  | 4296 | 1.394 | -0.0853 | No |
| 32 | CACNB1 |  |  | 4299 | 1.393 | -0.0807 | No |
| 33 | FMNL1 |  |  | 4315 | 1.384 | -0.0771 | No |
| 34 | ADAM11 |  |  | 4681 | 1.259 | -0.1006 | No |
| 35 | NKIRAS2 |  |  | 4766 | 1.231 | -0.1028 | No |
| 36 | PCTP |  |  | 4843 | 1.207 | -0.1044 | No |
| 37 | GFAP |  |  | 4853 | 1.205 | -0.1010 | No |
| 38 | AKAP1 |  |  | 4903 | 1.187 | -0.1007 | No |
| 39 | PPY |  |  | 5013 | 1.151 | -0.1051 | No |
| 40 | PSMC3IP |  |  | 5109 | 1.113 | -0.1085 | No |
| 41 | DBF4B |  |  | 5175 | 1.089 | -0.1097 | No |
| 42 | ANKRD40 |  |  | 5202 | 1.080 | -0.1080 | No |
| 43 | DDX52 |  |  | 5360 | 1.024 | -0.1165 | No |
| 44 | EZH1 |  |  | 5481 | 0.979 | -0.1222 | No |
| 45 | APPBP2 |  |  | 5497 | 0.975 | -0.1201 | No |
| 46 | TNS4 |  |  | 5589 | 0.948 | -0.1237 | No |
| 47 | KRT34 |  |  | 5681 | 0.918 | -0.1275 | No |
| 48 | KLHL11 |  |  | 5739 | 0.899 | -0.1288 | No |
| 49 | ABCA5 |  |  | 5786 | 0.882 | -0.1293 | No |
| 50 | SAMD14 |  |  | 5862 | 0.857 | -0.1321 | No |
| 51 | TBX4 |  |  | 5877 | 0.852 | -0.1302 | No |
| 52 | MLX |  |  | 5973 | 0.823 | -0.1346 | No |
| 53 | SOCS7 |  |  | 6024 | 0.802 | -0.1357 | No |
| 54 | WNT3 |  |  | 6241 | 0.732 | -0.1497 | No |
| 55 | TMEM92 |  |  | 6556 | 0.632 | -0.1714 | No |
| 56 | HOXB8 |  |  | 6757 | 0.570 | -0.1847 | No |
| 57 | RPL27 |  |  | 6815 | 0.546 | -0.1872 | No |
| 58 | RND2 |  |  | 6841 | 0.537 | -0.1872 | No |
| 59 | STAT3 |  |  | 6862 | 0.530 | -0.1869 | No |
| 60 | HAP1 |  |  | 6920 | 0.512 | -0.1895 | No |
| 61 | GOSR2 |  |  | 6978 | 0.496 | -0.1922 | No |
| 62 | THRAP4 |  |  | 7127 | 0.444 | -0.2019 | No |
| 63 | LOC90586 |  |  | 7191 | 0.421 | -0.2053 | No |
| 64 | GPATC8 |  |  | 7264 | 0.398 | -0.2094 | No |
| 65 | PNMT |  |  | 7325 | 0.374 | -0.2127 | No |
| 66 | MAP3K14 |  |  | 7392 | 0.347 | -0.2165 | No |
| 67 | KRT20 |  |  | 7412 | 0.342 | -0.2168 | No |
| 68 | PTRF |  |  | 7419 | 0.338 | -0.2161 | No |
| 69 | UTP18 |  |  | 7481 | 0.316 | -0.2197 | No |
| 70 | ABCC3 |  |  | 7506 | 0.306 | -0.2204 | No |
| 71 | GNGT2 |  |  | 7717 | 0.225 | -0.2357 | No |
| 72 | NMT1 |  |  | 7789 | 0.193 | -0.2404 | No |
| 73 | NME1 |  |  | 7807 | 0.187 | -0.2411 | No |
| 74 | HOXB6 |  |  | 7870 | 0.158 | -0.2452 | No |
| 75 | DCAKD |  |  | 7882 | 0.153 | -0.2456 | No |
| 76 | ARL17P1 |  |  | 7887 | 0.151 | -0.2453 | No |
| 77 | APR-2 |  |  | 8009 | 0.103 | -0.2542 | No |
| 78 | ATP5G1 |  |  | 8013 | 0.100 | -0.2541 | No |
| 79 | TBKBP1 |  |  | 8032 | 0.093 | -0.2551 | No |
| 80 | LRRC59 |  |  | 8052 | 0.087 | -0.2563 | No |
| 81 | GSDML |  |  | 8057 | 0.085 | -0.2563 | No |
| 82 | PYY |  |  | 8065 | 0.083 | -0.2566 | No |
| 83 | HOXB13 |  |  | 8160 | 0.052 | -0.2635 | No |
| 84 | NGFR |  |  | 8219 | 0.029 | -0.2678 | No |
| 85 | HOXB3 |  |  | 8265 | 0.011 | -0.2712 | No |
| 86 | CNTNAP1 |  |  | 8406 | -0.040 | -0.2818 | No |
| 87 | HCRT |  |  | 8744 | -0.186 | -0.3068 | No |
| 88 | EIF1 |  |  | 8748 | -0.187 | -0.3064 | No |
| 89 | PIP5K2B |  |  | 8769 | -0.196 | -0.3072 | No |
| 90 | BRCA1 |  |  | 8777 | -0.199 | -0.3071 | No |
| 91 | UBTF |  |  | 8820 | -0.219 | -0.3095 | No |
| 92 | SFRS1 |  |  | 8891 | -0.255 | -0.3140 | No |
| 93 | SMARCE1 |  |  | 9023 | -0.322 | -0.3228 | No |
| 94 | PLEKHM1 |  |  | 9102 | -0.363 | -0.3275 | No |
| 95 | DUSP3 |  |  | 9123 | -0.371 | -0.3278 | No |
| 96 | G6PC |  |  | 9124 | -0.372 | -0.3265 | No |
| 97 | CROP |  |  | 9177 | -0.392 | -0.3291 | No |
| 98 | HOXB7 |  |  | 9179 | -0.393 | -0.3279 | No |
| 99 | HDAC5 |  |  | 9206 | -0.408 | -0.3285 | No |
| 100 | SGCA |  |  | 9250 | -0.430 | -0.3303 | No |
| 101 | RPIP8 |  |  | 9287 | -0.450 | -0.3315 | No |
| 102 | AARSD1 |  |  | 9313 | -0.460 | -0.3318 | No |
| 103 | CBX1 |  |  | 9417 | -0.522 | -0.3379 | No |
| 104 | CA10 |  |  | 9516 | -0.570 | -0.3434 | No |
| 105 | SUZ12 |  |  | 9542 | -0.583 | -0.3433 | No |
| 106 | TUBG1 |  |  | 9551 | -0.592 | -0.3419 | No |
| 107 | WIRE |  |  | 9602 | -0.619 | -0.3436 | No |
| 108 | PDE6G |  |  | 9623 | -0.631 | -0.3429 | No |
| 109 | OSBPL7 |  |  | 9781 | -0.713 | -0.3524 | No |
| 110 | ATP6V0A1 |  |  | 9795 | -0.721 | -0.3510 | No |
| 111 | ACACA |  |  | 9816 | -0.735 | -0.3500 | No |
| 112 | PSMD3 |  |  | 9828 | -0.743 | -0.3483 | No |
| 113 | NBR2 |  |  | 9921 | -0.794 | -0.3526 | No |
| 114 | FKBP10 |  |  | 9973 | -0.827 | -0.3536 | No |
| 115 | PHB |  |  | 10007 | -0.852 | -0.3532 | No |
| 116 | PERLD1 |  |  | 10062 | -0.885 | -0.3543 | No |
| 117 | HOXB5 |  |  | 10165 | -0.938 | -0.3589 | No |
| 118 | PDK2 |  |  | 10207 | -0.962 | -0.3587 | No |
| 119 | MMP28 |  |  | 10335 | -1.053 | -0.3648 | No |
| 120 | ACLY |  |  | 10348 | -1.060 | -0.3621 | No |
| 121 | TMUB2 |  |  | 10577 | -1.224 | -0.3753 | No |
| 122 | GRN |  |  | 10630 | -1.268 | -0.3749 | No |
| 123 | FAM117A |  |  | 10781 | -1.375 | -0.3816 | Yes |
| 124 | MEOX1 |  |  | 10820 | -1.404 | -0.3797 | Yes |
| 125 | PLXDC1 |  |  | 10824 | -1.408 | -0.3751 | Yes |
| 126 | CCR10 |  |  | 10829 | -1.412 | -0.3706 | Yes |
| 127 | HEXIM1 |  |  | 10897 | -1.472 | -0.3707 | Yes |
| 128 | LOC284064 |  |  | 11067 | -1.629 | -0.3780 | Yes |
| 129 | LOC474170 |  |  | 11081 | -1.650 | -0.3733 | Yes |
| 130 | C17ORF73 |  |  | 11092 | -1.659 | -0.3684 | Yes |
| 131 | FZD2 |  |  | 11165 | -1.716 | -0.3680 | Yes |
| 132 | DDX5 |  |  | 11201 | -1.745 | -0.3647 | Yes |
| 133 | SC65 |  |  | 11226 | -1.765 | -0.3605 | Yes |
| 134 | SLC35B1 |  |  | 11337 | -1.865 | -0.3625 | Yes |
| 135 | SP2 |  |  | 11338 | -1.866 | -0.3562 | Yes |
| 136 | RSAD1 |  |  | 11397 | -1.930 | -0.3540 | Yes |
| 137 | AOC3 |  |  | 11448 | -1.988 | -0.3510 | Yes |
| 138 | JUP |  |  | 11461 | -1.998 | -0.3451 | Yes |
| 139 | CDK5RAP3 |  |  | 11467 | -2.003 | -0.3386 | Yes |
| 140 | VAT1 |  |  | 11561 | -2.096 | -0.3386 | Yes |
| 141 | KRT10 |  |  | 11580 | -2.108 | -0.3327 | Yes |
| 142 | HIGD1B |  |  | 11588 | -2.114 | -0.3260 | Yes |
| 143 | TUBG2 |  |  | 11638 | -2.153 | -0.3224 | Yes |
| 144 | ZNHIT3 |  |  | 11657 | -2.175 | -0.3163 | Yes |
| 145 | LGP2 |  |  | 11670 | -2.194 | -0.3098 | Yes |
| 146 | SNF8 |  |  | 11795 | -2.321 | -0.3113 | Yes |
| 147 | DHX8 |  |  | 11828 | -2.352 | -0.3057 | Yes |
| 148 | SKAP1 |  |  | 11833 | -2.353 | -0.2979 | Yes |
| 149 | EPN3 |  |  | 11835 | -2.355 | -0.2900 | Yes |
| 150 | MKS1 |  |  | 11870 | -2.402 | -0.2844 | Yes |
| 151 | MYST2 |  |  | 11938 | -2.484 | -0.2810 | Yes |
| 152 | NFE2L1 |  |  | 11977 | -2.534 | -0.2752 | Yes |
| 153 | SPOP |  |  | 11984 | -2.544 | -0.2670 | Yes |
| 154 | PLEKHH3 |  |  | 12133 | -2.750 | -0.2688 | Yes |
| 155 | SNX11 |  |  | 12162 | -2.795 | -0.2614 | Yes |
| 156 | ZNF403 |  |  | 12199 | -2.840 | -0.2545 | Yes |
| 157 | CHAD |  |  | 12230 | -2.889 | -0.2469 | Yes |
| 158 | UBE2Z |  |  | 12234 | -2.894 | -0.2372 | Yes |
| 159 | COPZ2 |  |  | 12238 | -2.897 | -0.2276 | Yes |
| 160 | ITGA3 |  |  | 12256 | -2.918 | -0.2189 | Yes |
| 161 | RAPGEFL1 |  |  | 12260 | -2.923 | -0.2091 | Yes |
| 162 | HOXB2 |  |  | 12310 | -2.977 | -0.2027 | Yes |
| 163 | IFI35 |  |  | 12324 | -2.993 | -0.1935 | Yes |
| 164 | FLJ20920 |  |  | 12373 | -3.057 | -0.1867 | Yes |
| 165 | SPATA20 |  |  | 12395 | -3.088 | -0.1777 | Yes |
| 166 | ZNF652 |  |  | 12454 | -3.182 | -0.1713 | Yes |
| 167 | ABCA6 |  |  | 12508 | -3.274 | -0.1641 | Yes |
| 168 | BECN1 |  |  | 12543 | -3.326 | -0.1553 | Yes |
| 169 | PEX12 |  |  | 12555 | -3.341 | -0.1448 | Yes |
| 170 | XYLT2 |  |  | 12564 | -3.363 | -0.1339 | Yes |
| 171 | PNPO |  |  | 12608 | -3.447 | -0.1254 | Yes |
| 172 | TOB1 |  |  | 12612 | -3.453 | -0.1138 | Yes |
| 173 | MAPT |  |  | 12626 | -3.480 | -0.1029 | Yes |
| 174 | ACBD4 |  |  | 12642 | -3.513 | -0.0921 | Yes |
| 175 | RAB5C |  |  | 12658 | -3.537 | -0.0811 | Yes |
| 176 | NSF |  |  | 12697 | -3.607 | -0.0717 | Yes |
| 177 | G6PC3 |  |  | 12710 | -3.639 | -0.0602 | Yes |
| 178 | SUPT4H1 |  |  | 12732 | -3.697 | -0.0491 | Yes |
| 179 | LOC339287 |  |  | 12802 | -3.894 | -0.0411 | Yes |
| 180 | LOC162427 |  |  | 12821 | -3.934 | -0.0290 | Yes |
| 181 | ATAD4 |  |  | 12892 | -4.116 | -0.0203 | Yes |
| 182 | CCDC56 |  |  | 13052 | -4.655 | -0.0165 | Yes |
| 183 | KRT19 |  |  | 13097 | -4.808 | -0.0034 | Yes |
| 184 | CALCOCO2 |  |  | 13249 | -5.950 | 0.0054 | Yes |
Table: GSEA details [plain text format]

  

Fig 2: CHR17Q21: Random ES distribution      
 Gene set null distribution of ES for **CHR17Q21**
